# Co-cultivation, Co-culture, Mixed Culture, and Microbial Consortium of Fungi: An Understudied Strategy for Biomass Conversion

**DOI:** 10.3389/fmicb.2021.837685

**Published:** 2022-01-20

**Authors:** Matheus Sanitá Lima, Rosymar Coutinho de Lucas

**Affiliations:** ^1^Department of Biology, University of Western Ontario, London, ON, Canada; ^2^Department of Biology, Faculty of Philosophy, Sciences and Letters of Ribeirão Preto, University of São Paulo, Ribeirão Preto, Brazil; ^3^Department of Parasitology, Microbiology and Immunology, Institute of Biological Sciences, Federal University of Juiz de Fora, Juiz de Fora, Brazil

**Keywords:** co-cultivation, co-culture, mixed culture, microbial consortium, microbial blend, biomass conversion, biorefinery, fungi

## Introduction

Sixty years have passed since Rachel Carson published her seminal book “Silent Spring” (Carson, 1962). Her work catapulted the ecological movement and shaped modern environmentalism (Kroll, 2001). However, fast-forward to present day and we seem not to have paid enough attention to the environment. Guidelines for a sustainable future have been repeatedly proposed and the planetary boundaries for our safe existence have been established (Rockström et al., 2009). Yet, more than 80% of the current global energy consumption still relies on unsustainable fossil fuels^1^ (Ritchie and Roser, 2020) and the COP26 negotiations have not delivered (Sheather, 2021). To make things worse, the demand for oil and gas is expected to peak in the next two decades.^2^ The prevailing linear economy based on the take-make-dispose system is unsustainable (Sariatli, 2017) and climate change already affects biological systems around the globe (Freitas et al., 2021). There will not be a “one-stop shop” type of solution, but we need to transition to a circular economy and biorefineries are a great place to start (Ubando et al., 2020). Among several models, the lignocellulosic biorefinery concept is prominent (Silva et al., 2018) and this is where fungi occupy a special place.

## Fungi—The Workhorse for the Production of Lignocellulolytic Enzymes

The importance of fungi for several industries is undeniable. These microorganisms produce enzymes that are used in a wide range of processes, from bread-making to paper manufacturing (Polizeli et al., 2005). But it is as producers of lignocellulolytic enzymes that fungi could be called a true workhorse. To put in (historical) perspective, the fungus *Trichoderma reesei* was first identified as a great cellulase producer over 75 years ago (Bischof et al., 2016). Since then, lignocellulolytic fungi have been studied to an unparalleled extent. Their enzymes have been characterized (Benassi et al., 2012), immobilized (da Silva et al., 2014), engineered (Furtado et al., 2015) and expressed in heterologous systems (Ribeiro et al., 2014). Several fungi have been screened for their lignocellulolytic capabilities (Benassi et al., 2014), and entire fungal genomes^1^,^2^ have been investigated in the search for holocellulose degrading pathways (Segato et al., 2014). Enzymatic cocktails derived from multiple fungi have been formulated as well (Pinheiro et al., 2021). However, the co-cultivation of fungi has lagged among the plethora of strategies for the production of lignocellulolytic enzymes. Broadly speaking, co-cultivation of microorganisms is the cultivation of two or more microbial strains combined within the same laboratory flask, Petri dish or fermentation tank. A co-cultivation can be referred also as a co-culture, mixed culture, mixed fermentation (more commonly used in submerged fermentation studies), microbial blend and microbial consortium. We use these terms interchangeably throughout the text, but in our discussion, we point to the potential advantages of having a standardized nomenclature. We have applied fungal co-cultures in biomass conversion studies (Sanitá Lima et al., 2016), and we now argue that there is a need (and opportunity) to take this strategy to a new level. Below, we identify some aspects that are missing in co-cultivation studies and present possible strategies for the community to move forward in this realm. These ideas stem from studies in fungal physiology, community ecology and synthetic biology. In fact, co-cultivation of microorganisms has long been applied in the investigation of natural products (Bertrand et al., 2014) and the development of synthetic biology techniques (Goers et al., 2014). Therefore, we believe greater cross-disciplinary discussions would enrich and spur strategies to produce fungal lignocellulolytic enzymes.

## The Metabolic Black-Box of Fungal Co-cultures

Fungi are extensively used in biotechnology, precisely because of their innate capacity to produce several proteins. As part of the fungal primary metabolism, lignocellulolytic enzymes are readily secreted according to growth conditions (de Lucas et al., 2021). But fungi have an intricate secondary metabolism and secrete numerous compounds into the culture medium (Frisvad, 2015). Although primary and secondary metabolites have been studied as separate entities, microorganisms know how to blurry our artificial classifications (Kistler and Broz, 2015). From day one, fungi produce compounds of diverse chemical nature that control spore germination, mycelial growth, clonal reproduction and defense (Leeder et al., 2011). Many of these molecules are density-dependent and act on quorum sensing (Albuquerque and Casadevall, 2012). Several other metabolites trigger the activation of silent gene clusters through elusive mechanisms of interspecies crosstalk (Marmann et al., 2014). This is how co-cultures quickly become a metabolic black-box. This is also where co-cultivation studies for the production of lignocellulolytic enzymes lack insight. Bacterial co-cultures, mostly referred as microbial/bacterial consortia, have been investigated to much greater detail and possess wider applications. In fact, bacterial consortia are fabricated for specific biotechnological goals (Vortmann et al., 2021). Synthetic microbial consortia borrow ecological concepts, such as amensalism and commensalism, to engineer high performance multi-species systems (Sgobba and Wendisch, 2020). Although co-cultivating fungi to produce better enzymatic cocktails is not a brand-new idea (Zoglowek et al., 2016), the studies within this domain fare poorly compared to their bacterial counterparts in terms of insight. Most experiments grow two to three strains under the same conditions used for the cultivation of one single strain (Sperandio and Filho, 2021). The effects of inoculum volume ratio (Rabello et al., 2014) and time (Kolasa et al., 2014) can be investigated, but this is not common place. So, secondary metabolites that can exert antagonistic effects are not taken into consideration and the co-cultivation performance (i.e., the final hydrolysis yield) is a result of trial-and-error. The co-cultures mostly have only fungi and use at least one strain that is known to be a good producer of biomass degrading enzymes (Wang et al., 2015). Several carbon and nitrogen sources are generally tested (Sperandio and Filho, 2019), but the effects of the fermentation style on the growing fungi are hard to tease apart in current set-ups.

Co-cultures have clear advantages over their axenic counterparts (Sperandio and Filho, 2019). Growing several strains altogether will reduce production costs, as inputs and human labor are better used. With the right strains, co-cultures can be more resistant to contamination and produce more powerful (i.e., synergistic) enzymatic cocktails (Gutiérrez-Correa and Villena, 2012). The emphasis here is on “right strains” and “synergistic” cocktails. Co-cultures tend to exhibit higher yields of biomass saccharification, but claims about multi-enzyme synergism can be loosely made. In fact, at the end of these experiments very little is known other than the final amount of reducing sugars released. Are the co-cultured (co-expressed?) enzymes acting synergistically or additively? What about the co-cultivation attempts that did not present higher yields of biomass hydrolysis? Have the co-cultured fungi inhibited each other via secondary metabolites or have the fungi run out of carbon source before producing all their enzymes? These are some questions that are commonly not present in most co-cultivation studies aiming to produce lignocellulolytic enzymes. The consequent lack of insight brought about by experimental design blind spots not only prevents the community from finding promising co-cultures, but also hinders the possibility of these systems being scaled-up. If experiments do not account for the inter-species metabolic talk happening inside an Erlenmeyer, how could these strategies be scaled up to industry applications?

We understand that these studies are focused on the production of cellulases, xylanases, and lignin-modifying enzymes. It is unfeasible to dissect every single co-culture using fully fledged metabolomics, proteomics, transcriptomics and epigenomics techniques. This is why we believe standardized group effort is the way to move forward. Co-cultivation systems represent a valuable (and untapped) source of multi-enzyme cocktails. We need cooperation across disciplines to make this strategy a successful approach.

## Discussion

What is in a name can create momentum around scientific findings and help spread concepts (Smith and James, 2013). In an attempt to strengthen the studies of lignocellulolytic co-cultures, our first suggestion is for researchers to adopt a common language. Currently, growing concomitantly several microorganisms for a specific purpose can be called a co-culture, mixed cultured, co-cultivation, mixed fermentation, microbial (e.g., fungal) consortium, microbial cocktail, and microbial blend. This list is not comprehensive and name variants exist depending on the applications of the study. Although certain name choices hold intrinsic value according to different fields, having a cross-disciplinary nomenclature can help with scientific dissemination and galvanize collaborations. In fact, we are not the first ones to highlight the need for standardized names. Del Frari and Ferreira (2021) have proposed the term “skopobiota” to move forward, for instance.

Our other suggestion is the creation of a database of co-cultures. Databases have been fundamental to data-rich research endeavors in molecular evolution (Smith and Sanitá Lima, 2017) and microbial community ecology (Sanitá Lima et al., 2019). However, databases must be standardized and possibly curated for them to hold meaning and value in springing future research (Sanitá Lima and Smith, 2017). As researchers start to adopt a common nomenclature, each co-cultivation assay could be stored in this “database of co-cultures.” Experimental variables, such as cultivation conditions, and number and name of strains, could be standard entries that would be easily retrieved for future reference and comparative analyses. Each combination of fungi, pairwise or not, could be classified according to their ecological interactions—whether there was commensalism, mutualism, antagonism, etc. Data pertaining to metabolic, proteomic, transcriptomic and epigenomic analyses could be added. This would serve as a roadmap to indicate knowledge gaps and possible points of reference—the species X with commensal Y produced metabolite Z after W days. As data are gathered, the database could give rise to a mix-and-match system through which future co-cultures would be more easily designed and enzymatic cocktails optimized. This approach resembles the prolific tinkering of other systems, such as the OSMAC approach (Bode et al., 2002) and GENPLAT platform (Banerjee et al., 2010). The possibilities are endless, just like the potentialities of the co-cultivation of lignocellulolytic fungi.

The current costs for the production of lignocellulosic biofuels are still mostly prohibitive (Rosales-Calderon and Arantes, 2019). Great part of these costs derives from the biomass pretreatment and production of enzymes for biomass saccharification (de Oliveira Gorgulho Silva and Filho, 2017). Co-cultivation of lignocellulolytic fungi can produce cheaper enzymes and make biomass conversation technologies more feasible. Studying fungal enzymes has spawned profitable industries and decades-long research programs. These enzymes will not save the world, but can certainly contribute to a less wasteful one. The transition from a linear present to a circular future is a true maze, and we need to start from somewhere. Otherwise, we risk not only having a silent spring, but a silent planet.

## Author Contributions

MS and RC conceptualized the idea, investigated pertinent literature, discussed concepts, and wrote the manuscript. All authors approved the final version.

## Funding

Previous research that spawned this work has been funded by the National Council for Scientific and Technological Development (CNPq) and Fundação de Amparo à Pesquisa do Estado de São Paulo (FAPESP).

## Conflict of Interest

The authors declare that the research was conducted in the absence of any commercial or financial relationships that could be construed as a potential conflict of interest.

## Publisher's Note

All claims expressed in this article are solely those of the authors and do not necessarily represent those of their affiliated organizations, or those of the publisher, the editors and the reviewers. Any product that may be evaluated in this article, or claim that may be made by its manufacturer, is not guaranteed or endorsed by the publisher.

## Footnotes

^1^ https://www.bp.com/en/global/corporate/energy-economics/statistical-review-of-world-energy.html

^2^ https://www.mckinsey.com/~/media/McKinsey/Industries/Oil%20and%20Gas/Our%20Insights/Global%20Energy%20Perspective%202021/Global-Energy-Perspective-2021-final.pdf
